# Nickel oxide nanoparticles can recruit eosinophils in the lungs of rats by the direct release of intracellular eotaxin

**DOI:** 10.1186/s12989-016-0142-8

**Published:** 2016-06-09

**Authors:** Seonghan Lee, Sung-Hyun Hwang, Jiyoung Jeong, Youngju Han, Sung-Hyun Kim, Dong-Keon Lee, Hae-Suk Lee, Seung-Tae Chung, Jayoung Jeong, Changhyun Roh, Yun Seok Huh, Wan-Seob Cho

**Affiliations:** 1 Lab of Toxicology, Department of Medicinal Biotechnology, College of Health Sciences, Dong-A University, 840 Hadan 2dong, Saha-gu, Busan, 604-714 Republic of Korea; 2 Busan IL Science High School, Busan, Republic of Korea; 3 Division of Toxicological Research, National Institute of Food and Drug Safety Evaluation, Ministry of Food and Drug Safety, Osong, Republic of Korea; 4 Division of Biotechnology Research, Advanced Radiation Technology Institute, Korea Atomic Energy Research Institute, Jeonbuk, Republic of Korea; 5 Department of Biological Engineering, Biohybrid Systems Research Center, Inha University, Incheon, Republic of Korea

**Keywords:** Nickel oxide nanoparticles, Solubility, Cytotoxicity, Eotaxin, Eosinophil

## Abstract

**Background:**

Instillation of highly soluble nanoparticles (NPs) into the lungs of rodents can cause acute eosinophilia without any previous sensitizations by the role of dissolved ions. However, whether gradually dissolving NPs can cause the same type of eosinophilia remains to be elucidated. We selected nickel oxide (NiO) as a gradually dissolving NP and evaluated the time course pulmonary inflammation pattern as well as its mechanisms.

**Methods:**

NiO NPs were intratracheally instilled into female Wistar rats at various concentrations (50, 100, and 200 cm^2^/rat) and the lung inflammation was evaluated at various time-points (1, 2, 3, and 4 days). As positive controls, NiCl_2_ and the ovalbumin-induced allergic airway inflammation model was applied. NiCl_2_ was instilled at 171.1 μg Ni/rat, which is equivalent nickel concentration of 200 cm^2^/rat of NiO NPs. Cytological analysis and biochemical analysis including lactate dehydrogenase (LDH), total protein, and pro-inflammatory cytokines were measured in bronchoalveolar lavage fluid (BALF). The levels of total immunoglobulin E (IgE) and anaphylatoxins (C3a and C5a) were measured in BALF and serum. The levels of eotaxin were measured in the alveolar macrophages and normal lung tissue before and after addition of cell lysis buffer to evaluate whether the direct lysis of cells can release intracellular eotaxin.

**Results:**

NiO NPs produced acute neutrophilic inflammation throughout the study. However, eosinophils were recruited at 3 and 4 days post-instillation of NiO NPs and the magnitude and pattern of inflammation was similar with NiCl_2_ at 24 h post-instillation. The eosinophil recruitment by NiO NPs was not related with either the levels of total IgE or anaphylatoxins. The lysis of alveolar macrophages and normal lung tissue showed high levels of intracellular eotaxin and the levels of LDH showed positive correlation with the levels of eotaxin.

**Conclusions:**

Instillation of NiO NPs produced neutrophilia at 1 and 2 days after instillation, while the mixed type of neutrophilic and eosinophilic inflammation was produced at 3 and 4 days post-instillation, which was consistent with NiCl_2_. The mechanism of the eosinophilia involves the direct release of intracellular eotaxin due to the rupture of cells by the accumulated solubilized nickel ions in the phagolysosome.

**Electronic supplementary material:**

The online version of this article (doi:10.1186/s12989-016-0142-8) contains supplementary material, which is available to authorized users.

## Background

Several mechanisms have been known to cause eosinophilic inflammation including the immunoglobulin E (IgE)-mediated allergic response as well as anaphylatoxins by the complement activation cascade. IgE-mediated eosinophilia, commonly known as an allergic reaction, can be produced by an immunological response synchronized by several sensitization processes against a certain allergen [1] or parasite infection [2]. The activation of complement releases anaphylatoxins (e.g., C3a and C5a) and produces eosinophilia by histamine release from mast cells. This type of inflammation is generally called as pseudoallergy or drug hypersensitivity [3]. In our recent studies, however, some metal oxide nanoparticles (NPs) were found to cause pulmonary eosinophilia within 24 h without any previous sensitization [4]. Although previous studies showed that instillation of TiO_2_ promotes allergic sensitization and lung inflammation in the ovalbumin (OVA)-sensitization mouse model [5], and that liposome-based NPs produced complement-mediated hypersensitivity [6], the mechanism of eosinophilia by metal oxide NPs (e.g., CoO, CuO, and ZnO) within 24 h without any previous sensitization is new finding that deserves further investigation [7–9].

Acute eosinophil infiltration caused by metal oxide NPs has been reported with highly soluble NPs; the dissolved metal ions in the acidic phagolysosomes of phagocytes play a critical role in recruiting eosinophils. When highly soluble NPs are inhaled, NPs remain insoluble in the alveoli, but are dissolved soon after phagocytosis, releasing their compositional ions [10]. Indeed, several highly soluble NPs produced acute eosinophilic airway inflammation in rodents via their soluble metal ions [7, 9]. The molecular mechanism of eosinophilia by NPs involves the direct induction of the eotaxin protein; however, the signaling pathway for eotaxin gene expression is still poorly understood [11]. Furthermore, other possible mechanisms for eosinophil infiltration by metal oxide NPs (e.g., IgE-mediated or anaphylatoxins by complement activation) are still in question.

Solubility is one of the key issues in triggering the eosinophilic inflammation of metal oxide NPs. All NPs known to produce eosinophilia in the lung are highly soluble NPs (e.g., CoO, CuO, and ZnO), but whether gradually dissolving NPs can produce eosinophilia is not known. Therefore, follow-on our previous study, we hypothesized, in this study, that gradually dissolving NPs can produce pulmonary eosinophilia by accumulating solubilized ions in phagocytes with a delayed time frame as compared with highly soluble NPs. We selected NiO NPs as a representative gradually dissolving NP based on our previous study [12], and investigated the time course of inflammation and its underlying mechanism using a rat intratracheal instillation model.

## Results

### Physicochemical characterization of NiO NPs

Physicochemical properties of NiO NPs are summarized in Table 1. Transmission electron microscopy (TEM) and field emission-type scanning electron microscopy (FE-SEM) analysis showed that NiO NPs were spherically shaped and the average diameter of NiO NPs was about 5.3 nm (Figure S1, see Additional file 1). NiO NPs formed large agglomerates when dispersed in phosphate buffered saline (PBS). However, 3 % rat serum improved the dispersion, such that it became similar to that of in distilled water (DW). The zeta potential of NiO NPs was positive in DW, but negative in PBS or PBS with 3 % rat serum. NiO NPs showed 9.5 % and 1.1 % dissolution within 24 h in artificial lysosomal fluid and PBS, respectively. Incubation of NiO NPs in PBS with 3 % rat serum showed about 2.2 % dissolution, which was slightly higher than that of without serum. NiO NPs showed no endotoxin contamination.

**Table 1:** Physicochemical characterization of NiO NPs

Measurements	Mean ± SEM
Primary size (nm)	5.3 ± 0.4
Surface area (m^2^/g)	91.8
Hydrodynamic size (nm) in	
DW	209 ± 3.7
PBS	970 ± 52
PBS with 3 % rat serum	224 ± 11
Polydispersity in	
DW	0.30 ± 0.02
PBS	0.65 ± 0.05
PBS with 3 % rat serum	0.45 ± 0.06
Zeta potential (mV) in	
DW	48.9 ± 0.6
PBS	−21 ± 0.6
PBS with 3 % rat serum	−30 ± 2.3
Solubility (%) in	
PBS (pH 7.4)	1.1
PBS with 3 % rat serum	2.2
Artificial lysosomal fluid (pH 5.5)	9.5
Endotoxin	<0.1 EU/mL

### Pulmonary inflammation pattern evaluated by differential cell counts of BALF

**Fig. 1:**
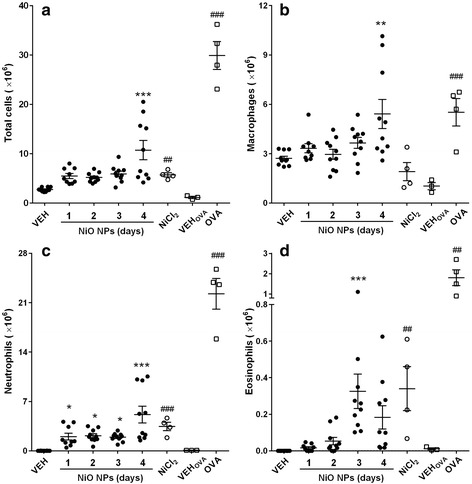
Cytological analysis of BALF after intratracheal instillation of NiO NPs, NiCl_2_, and OVA. NiO NPs were instilled at 200 cm^2^/rat and cytological evaluation was performed at 1, 2, 3, and 4 days after instillation. NiCl_2_ was instilled at 378.0 μg/rat, which is equivalent to the nickel dose (171.1 μg Ni/rat) of NiO NPs at 200 cm^2^/rat, and cytological evaluation was performed at 1 day after instillation. **a**, Number of total cells. **b** Number of macrophages. **c** Number of neutrophils. **d** Number of eosinophils. Abbreviations: VEH, vehicle control for NiO NPs and NiCl_2_; OVA, ovalbumin-induced allergic airway inflammation; VEH_OVA_, vehicle control for OVA group. Mean ± SEM (*n* = 10 for NiO NPs and *n* = 4 for NiCl_2_ and OVA group). One-way ANOVA test was applied for comparison between NiO NPs and VEH with statistical significance indicated by ^*^ *p* < 0.05, ^**^ *p* < 0.01, and ^***^ *p* < 0.001. Student’s *t*-test was applied for comparison of NiCl_2_ against VEH and OVA group against VEH_OVA_ with statistical significance indicated by ^##^ *p* < 0.01 and ^###^ *p* < 0.001

To evaluate the time-course inflammation pattern of NiO NPs, single intratracheal instillation was performed at concentrations of 50, 100, and 200 cm^2^/rat which corresponding 54.5, 109, and 218 μg/rat in a mass basis and the type and magnitude of lung inflammation was evaluated at various time-points. As a positive control for dissolved nickel ions, NiCl_2_ was instilled at 171.1 μg Ni/rat, which is equivalent nickel concentration of 200 cm^2^/rat of NiO NPs. The OVA-induced airway inflammation model was established as a positive control for IgE-mediated eosinophilic inflammation. To confirm that the unique inflammation pattern by NiO NPs is not just a coincidence, 10 rats per group were used for the evaluation of time-course inflammation of NiO NPs at 200 cm^2^/rat, while other experiments were performed with 4 rats per group. Data for the cytological analysis of bronchoalveolar lavage fluid (BALF) is presented in Fig. 1. NiO NPs (200 cm^2^/rat) at 4 days after instillation showed higher number of total cells, macrophages, and neutrophils compared to other time-points which might be due to the continuous stimulation by NPs as well as dissolved ions. This worsening inflammation pattern is also consistent with the cytological data of NiO NPs at 50 and 100 cm^2^/rat (Figure S2, see Additional file 1). Although the numbers of inflammatory cells showed statistical significance compared to vehicle control, the values were much less than those of 200 cm^2^/rats. While, NiO NPs at 200 cm^2^/rat significantly increased the number of neutrophils at all time points, ranging from about 30 to 50 % of total cells. The number of eosinophils significantly increased at 3 days after instillation that was about 5.5 % of total cells. The lymphocytes in BALF showed no significant increases and the percentage of lymphocytes was less than 0.5 % and basophils were not observed in any treatment groups (data not shown). The type and magnitude of inflammation by NiCl_2_ at 24 h after instillation were very similar to that of high dose group of NiO NPs at 3 and 4 days after instillation. The OVA-induced airway inflammation model showed significant increases in the number of neutrophils and eosinophils in BALF at 24 h after challenge.

### Levels of Ni in BALF

The levels of Ni in BALF of 200 cm^2^/rat of NiO NPs showed significant increases at all time-points (Figure S3, see Additional file 1). The levels of Ni were peaked at 1 or 2 days after instillation and showed slightly decreasing trend thereafter.

### Levels of lactate dehydrogenase (LDH) and total protein in BALF

**Fig. 2:**
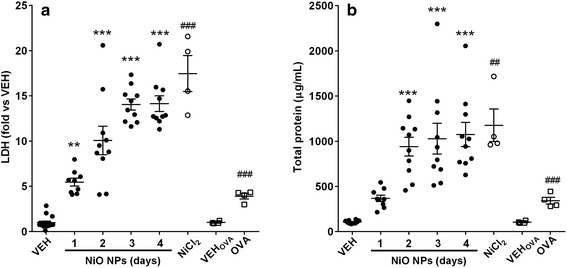
Levels of lactate dehydrogenase (LDH) and total protein in BALF treated with NiO NPs, NiCl_2_, and OVA. NiO NPs were instilled at 200 cm^2^/rat and cytological evaluation was performed at 1, 2, 3, and 4 days after instillation. NiCl_2_ was instilled at 378.0 μg/rat, which is equivalent to the nickel dose (171.1 μg Ni/rat) of NiO NPs at 200 cm^2^/rat, and cytological evaluation was performed at 1 day after instillation. **a** Levels of LDH. **b**, Levels of total protein. Abbreviations: VEH, vehicle control for NiO NPs and NiCl_2_; OVA, ovalbumin-induced allergic airway inflammation; VEH_OVA_, vehicle control for OVA group. Mean ± SEM (*n* = 10 for NiO NPs and *n* = 4 for NiCl_2_ and OVA group). One-way ANOVA test was applied for comparison between NiO NPs and VEH with statistical significance indicated by ^**^ *p* < 0.01 and ^***^ *p* < 0.001. Student’s *t*-test was applied for comparison of NiCl_2_ against VEH and OVA group against VEH_OVA_ with statistical significance indicated by ^##^ *p* < 0.01 and ^###^ *p* < 0.001

The levels of LDH and total protein at 200 cm^2^/rat of NiO NPs were significant increased 2–4 days after instillation (Fig. 2). The levels of LDH and total protein at 50 and 100 cm^2^/rat of NiO NPs were increased with dose-dependent and time-dependent manner (Figure S4, see Additional file 1). Although significant increases were found at 50 and 100 cm^2^/rats, these values were much less than those of 200 cm^2^/rats. Instillation of NiCl_2_ produced similar magnitude of increases in the levels of LDH and total protein of 200 cm^2^/rat of NiO NPs at 3 and 4 days after instillation (Fig. 2). The OVA-sensitized group showed significant increases in the levels of LDH and total protein; however, the magnitude of the increase was much smaller than that produced by the high dose of NiO NPs (Fig. 2).

### Expression of pro-inflammatory cytokines in BALF

**Fig. 3:**
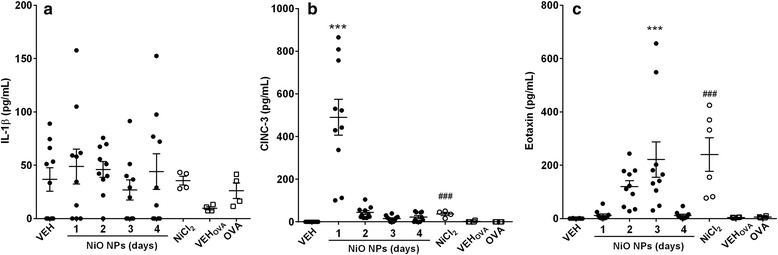
Pro-inflammatory cytokines in BALF after intratracheal instillation of NiO NPs, NiCl_2_, and OVA. NiO NPs were instilled at 200 cm^2^/rat and evaluated the expression of pro-inflammatory cytokines at 1, 2, 3, and 4 days after instillation. NiCl_2_ was instilled at 378.0 μg/rat, which is equivalent to the nickel dose (171.1 μg Ni/rat) of NiO NPs at 200 cm^2^/rat, and pro-inflammatory cytokines were measured at 1 day after instillation. **a** IL-1β. **b** CINC-3. **c** Eotaxin. Abbreviations: VEH, vehicle control for NiO NPs and NiCl_2_; OVA, ovalbumin-induced allergic airway inflammation; VEH_OVA_, vehicle control for OVA group. Mean ± SEM (*n* = 10 for NiO NPs and *n* = 4 for NiCl_2_ and OVA group). One-way ANOVA test was applied for comparison between NiO NPs and VEH with statistical significance indicated by ^***^ *p* < 0.001. Student’s *t*-test was applied for comparison of NiCl_2_ against VEH and OVA group against VEH_OVA_ with statistical significance indicated by ^###^ *p* < 0.001

Data for pro-inflammatory cytokines in BALF is presented in Fig. 3. The concentrations of interleukin (IL)-1β were not significantly increased at any time points, while the levels of cytokine-induced neutrophil chemoattractant-3 (CINC-3) significantly increased at 1 day after instillation of NiO NPs at 200 cm^2^/rat. The concentrations of eotaxin significantly increased at 3 days after instillation of NiO NPs at 200 cm^2^/rat. However, the levels of IL-4 and interferon-γ (IFN-γ) in the BALF of any treatment group were below the detection limit (15.6 pg/mL for IL-4 and 39.1 pg/mL for IFN-γ) (data not shown). Instillation of NiCl_2_ produced significant increases of the levels of CINC-3 and eotaxin, while the levels of IL-4 and IFN-γ were below the detection limit (data not shown). OVA-sensitized rats showed no significant increases in IL-1β, IL-4, CINC-3, IFN-γ, and eotaxin (data not shown for IL-4 and IFN-γ).

### Levels of total IgE and anaphylatoxins (C3a and C5a) in serum and BALF

**Fig. 4:**
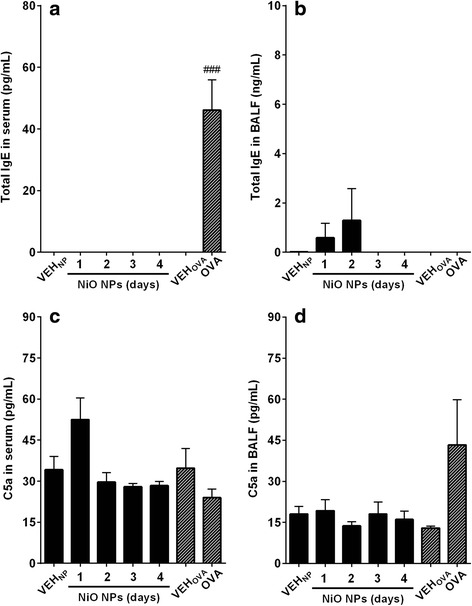
Measurement of total IgE and C5a in serum or BALF after intratracheal instillation of NiO NPs at 200 cm^2^/rat. Total IgE levels were measured in (**a**) serum and (**b**) BALF. Levels of C5a were measured in (**c**) serum and (**d**) BALF. Abbreviations: VEH_NP_, vehicle control for NiO NPs; OVA, ovalbumin-induced allergic airway inflammation; VEH_OVA_, vehicle control for OVA group. Mean ± SEM (*n* = 4). Student’s *t*-test was applied for comparison between OVA group and vehicle control (VEH_OVA_) with statistical significance indicated by ^###^ *p* < 0.001

The levels of total IgE and anaphylatoxins (C3a and C5a) were measured because these molecules underlie typical mechanisms for eosinophil recruitment. The levels of total IgE in serum significantly increased only in the OVA-sensitization group, while other groups showed no significant changes in serum or BALF (Fig. 4). The levels of C5a in any treatment group including the OVA-sensitization group showed no significant changes as compared to the vehicle control (Fig. 4). The levels of C3a in all treatment groups was the under the detection limit (0.625 ng/mL) (data not shown).

### Levels of intracellular eotaxin in alveolar macrophages and normal lung tissue

**Fig. 5:**
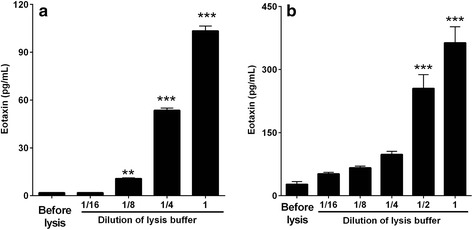
Levels of eotaxin in alveolar macrophages or normal lung tissue before/after incubation with lysis buffer. Alveolar macrophages and normal lung tissue were incubated with different dilutions of cell lysis buffer at 1 × 10^7^ cells/mL for alveolar macrophages and 50 mg/mL for normal lung tissue, and the levels of eotaxin in the supernatants were measured. Lysis of (**a**) alveolar macrophages and (**b**) normal lung tissue with undiluted cell lysis buffer showed massive increases in the levels of eotaxin and serial dilution of lysis buffer showed a good dose–response. While samples before the addition of cell lysis buffer showed minimal levels of eotaxin. Mean ± SEM (*n* = 4). One-way ANOVA test was applied for comparison of treated groups against control (samples before lysis) with statistical significance indicated by ^**^ *p* < 0.01 and ^***^ *p* < 0.001

To evaluate the mechanism of eotaxin release by NiO NPs, the levels of intracellular eotaxin were measured in alveolar macrophages and normal lung tissue. Lysis of 1 × 10^7^ alveolar macrophages using 1 mL of undiluted cell lysis buffer produced about 100 pg/mL of eotaxin and serial dilution of cell lysis buffer showed a good dose–response (Fig. 5a). Lysis of 50 mg of normal lung tissue using 1 mL of undiluted cell lysis buffer produced about 350 pg/mL of eotaxin and serial dilution of lysis buffer showed a good dose–response (Fig. 5b). The detected eotaxin was considered intracellular eotaxin because cells or tissues before lysis demonstrated minimal levels of eotaxin.

### Positive correlation between levels of LDH and eotaxin

**Fig. 6:**
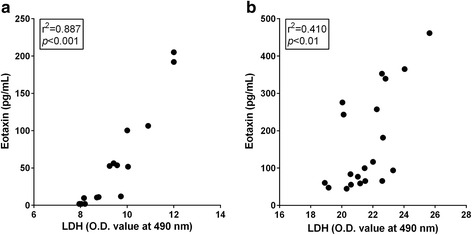
Correlation between LDH and eotaxin. The levels of LDH in alveolar macrophages or normal lung tissue after incubation with lysis buffer were plotted against the levels of eotaxin measured in the same sample. **a** Alveolar macrophages. **b** Normal lung tissue. The levels of LDH demonstrated a positive trend with the levels of eotaxin with an excellent correlation coefficient. Pearson correlation test was applied

To evaluate the role of intracellular eotaxin by direct rupture of cells on acute eosinophilic inflammation, the levels of LDH in alveolar macrophages or normal lung tissue after incubation with lysis buffer were plotted against the levels of eotaxin measured in the same sample. The levels of LDH, the marker for cytotoxicity, showed positive correlation with the levels of eotaxin in alveolar macrophages as well as in normal lung tissue (Fig. 6). The Pearson correlation test in alveolar macrophages showed better correlation coefficient (*r* ^*2*^ = 0.887, *p* < 0.001) than that of normal lung tissue (*r* ^*2*^ = 0.410, *p* < 0.01).

## Discussion

To our knowledge, NiO NPs are gradually dissolving NPs that release a significant amount of nickel ions into the lysosomes of phagocytes and require more than 1 month for complete dissolution [12]. Previous studies on the pulmonary exposure of NiO NPs reported that NiO NPs in the lung produced acute neutrophilic inflammation via surface reactivity such as the generation of reactive oxygen species (ROS) [13, 14], while chronic active pulmonary inflammation with immunological T helper type 1 (Th1-type) hypersensitivity was produced by the chronic coexistence of NPs and haptenic soluble nickel ions [12]. In this study, we found that the instillation of NiO NPs produced massive eosinophilic inflammation during the period of acute neutrophilic inflammation. The various inflammatory patterns by NiO NPs including neutrophilia, eosinophilia, and chronic active/Th-1 hypersensitivity is very unique and has not been reported yet by any materials.

The eosinophilia of NiO NPs with a delayed-time frame (3 and 4 days after instillation) and immediate (1 day after instillation) eosinophilia by NiCl_2_ might provide an important clue for the mechanism of the immediate eotaxin release by highly soluble NPs (e.g., CoO, CuO, and ZnO) [7, 8]. Because eotaxin is stored in the cytoplasm of various normal cells such as the epithelial cell, fibroblast, and macrophage [15, 16], the lysis of these cells can release a significant amount of eotaxin protein. The delayed time frame of eosinophilia by NiO NPs found in this study might be due to the delayed rupture of cells by the accumulation of a significant amount of nickel ions from the NiO NPs in the phagolysosomes, because NiO NPs showed about 9.5 % dissolution/day in the acidic artificial lysosomal fluid. The increased levels of nickel in BALF measured in this study also confirm the dissolved nickel ions from NiO NPs. The role of nickel ions on recruiting eosinophils is supported not only by the increased levels of eotaxin at 1 day after instillation of NiCl_2_ which observed in this study, but also by the increased eotaxin levels of the highly soluble NPs (CoO, CuO, and ZnO) and their composition ions (CoCl_2_ and ZnCl_2_) at 1 day after instillation in our previous studies [8, 11]. No reports about the direct induction of eosinophilia by poorly soluble NPs without any previous sensitizations and the correlation plot of the levels of LDH, a marker for cell lysis, against the levels of eotaxin that was shown in this study also confirm this hypothesis. Therefore, this study can suggest one of main mechanisms of pulmonary eosinophilia by metal oxide NPs is the release of intracellular eotaxin from ruptured cells, following the accumulation of highly toxic soluble ions.

Eotaxin-1 (CCL11), which measured in this study is a potent chemoattractant for eosinophils and has been suggested as a therapeutic target as well as biomarker in various allergic diseases including asthma [17]. Eotaxin is an eosinophil-selective chemokine and not responsible to neutrophils and monocytes because those cells are not expressing CCL11-specific ligands including CCR2, CCR3, and CCR5 [18]. Eotaxin also regulates the baseline level of eosinophils in healthy status by constitutively expressing mRNA and/or protein in normal cells such as fibroblast, epithelial cells, endothelial cells, T cells, and macrophages [19–21]. Among various cell types, eosinophils possess much higher amount of eotaxin in matrix of crystalloid granules and release eotaxin by the activation of eosinophils [21]. Therefore, the eosinophilia by NiO NPs found in this study might be firstly triggered by the directly released intracellular eotaxin by the rupture of phagocytic cells and then recruited eosinophils accelerate eosinophilia. However, further studies are needed to support this hypothetical mechanism of action.

While the direct induction of eotaxin was shown in this study, the involvement of IgE or complement activation by metal oxide NPs in the pulmonary eosinophilia was unknown. Previous studies showed that various metal oxide NPs (e.g., Fe_2_O_3_, TiO_2_, and ZnO) and carbon nanotubes have an adjuvant effect, promoting allergic airway inflammation in the OVA-sensitization mouse model [5, 22–24]. Because IgE-mediated airway inflammation essentially requires an allergenic epitope and several sensitization processes, the direct induction of IgE-response by metal oxide NPs in an acute phase without previous sensitizations is highly unlikely. The lack of significant increases in IgE and IL-4 levels in this study also confirms that the eosinophilia by NiO NPs was not produced by the Th2 response. However, the low levels of eotaxin in the OVA model found in this study might be due to the pattern of eotaxin expression is transient [25]. The release of anaphylatoxins such as C3a and C5a via the complement cascade can also produce acute eosinophilia; previous studies showed that some micelles, liposomes, and NPs produce an acute allergic response [3, 26, 27]. However, those studies were performed not in inhalation models, but in intravenous injection models or in vitro systems using serum. No significant changes in C3a and C5a levels found in this study might imply that NiO NPs does not stimulate anaphylatoxins by complement activation in this experimental setting. However, a slightly increased levels of serum C5a at 1 day after instillation and the low group size cannot exclude that some effects on C5a.

The mechanism of eosinophilia by NiO NPs at day 3 and 4 after instillation might provide important information for the asthmatic lung disease induced by environmental particles such as diesel exhaust particles and particulate matter 2.5 (PM2.5). Indeed, several studies showed that increased human exposure to diesel exhaust particles and PM is related to increased asthma incidence [28, 29]. However, the exact mechanism for the development of the asthmatic lung by air pollution is still unknown, partly because these particles are complex and composed of multiple particle types and organic compounds. Epidemiological data suggests that asthma develops not only after sensitization with allergens including food allergens [30], but also by a non-allergic mechanism such as polycyclic aromatic hydrocarbons [31]. Based on interesting literature on the zinc level in the PM with a positive trend with asthma morbidity [32], the direct induction of eotaxin protein by NiO NPs without involvement of IgE or complement activation can suggest one possible mechanism for this non-allergic asthmatic lung injury by PM and further studies are warranted.

## Conclusion

Intratracheal instillation of NiO NPs in rats demonstrated a unique mixed type of neutrophilic and eosinophilic inflammation at 3 and 4 days after instillation, which was consistent with the inflammation by NiCl_2_ at 1 day after instillation. The mechanism of eosinophilia by NiO NPs is due to the involvement of the direct rupture of cells, releasing a significant level of intracellular eotaxin. In addition, the time frame for eosinophilia was variable depending on the solubility of NPs and the intrinsic toxicity of compositional metals. However, more studies are warranted to support this hypothetical mechanism of eosinophilia by metal oxide NPs.

## Methods

### Nanoparticles and characterization

NiO NPs were purchased from Nanostructured & Amorphous Materials, Inc. (Houston, TX, USA). Physicochemical characterization including surface area, primary size, hydrodynamic size, polydispersity, zeta potential, solubility, and levels of endotoxin contamination were determined. Briefly, the surface area was measuring using the BET (Brunauer, Emmett, and Teller) method in ParticleCIC Ltd. (Leeds, UK) with a Micromeritics TriStar 3000 analyzer (Micromeritics Ltd., Bedfordshire, UK). The shape and primary size of the NiO NPs was measured using TEM (JEM-1200EX II, JEOL, Tokyo, Japan) and FE-SEM (Hitachi, Tokyo, Japan) and the average size was calculated by measuring at least 200 separate particles using a built-in program (JEOL). The hydrodynamic size and zeta potential of NiO NPs in DW or in PBS with/without dispersion medium (3 % heat-activated rat serum) was measured by Zetasizer Nano-ZS (Malvern, Malvern Hill, UK) according to the manufacturer’s instruction. To measure the solubility of the NiO NPs, NPs were suspended in various conditions including PBS (pH 7.4), PBS with 3 % rat serum, and artificial lysosomal fluid (pH 5.5) [33] at 1 mg/mL and incubated for 24 h at room temperature with continuous stirring. The NP-free supernatants were collected by three rounds of centrifugation at 15000 g for 30 min and the absence of NPs were confirmed by dynamic light scattering using Zetasizer Nano-ZS (Malvern). The concentration of dissolved metal ions was measured by the Center for Collaborative Instruments at Dong-A University using inductively coupled mass spectrometry (ICP-MS; Agilent Technologies, Seoul, Korea). Solubility was calculated and expressed as a percentage of the detected mass of nickel over the initial mass of nickel in NiO NPs. The concentration of endotoxin of NiO NPs at 400 cm^2^/mL in sterile PBS, the equivalent dose for animal experimentation, was evaluated by an endpoint chromogenic *Limulus* Amoebocyte Lysate (LAL) assay kit (Cambrex, Walkersville, MD, USA) with a detection limit of 0.1 EU/mL.

### Dispersion of NPs for animal experiment

Previous studies showed that NPs are tend to agglomerate when dispersed in medium and agglomerations are accelerated in high salt conditions such as PBS and saline [34]. Therefore, we used serum protein as a dispersion medium to provide protein corona formation, which can improve dispersion of NPs in physiological condition as previously described [34]. Briefly, the stock solution of NiO NPs was prepared by dispersion at 4000 cm^2^/mL (4360 μg/mL) in DW. The solution was sonicated for 10 min using a bath sonicator (Saehan-Sonic, Seoul, Korea) and heat-inactivated rat serum collected from healthy 6-week old female Wistar rats (Samtako, Gyeonggi-do, Korea) was added at 3 % of end concentration and sonicated for 5 min. Finally, Ca^2+^- and Mg^2+^-free PBS (Life Technologies, Gaithersburg, MD, USA) was added to the NP suspension to achieve pre-determined final concentrations (100, 200, and 400 cm^2^/mL) for animal experimentation, followed by sonication for 5 min in a bath sonicator (Saehan-Sonic).

### Intratracheal instillation of NiO NPs

Animal protocols were reviewed and approved by the Institutional Animal Care and Use Committee in Dong-A University. Six-week old female Wistar rats (Samtako) were acclimatized for 7 days before experimentation. Rats were housed in an individually ventilated cage system with controlled temperature and humidity (22 ± 1 °C and 50 ± 10 %) with a 12 h light/dark cycle. NiO NP suspensions were intratracheally instilled into the lungs of rats at surface area doses of 50, 100, and 200 cm^2^/rat (54.5, 109, and 218 μg/rat) according to the previously described method [8]. Briefly, rats were anesthetized by isoflurane inhalation (Piramal Critical Care, Bethlehem, PA, USA) using a rodent anesthesia system (VetEquip, Pleasanton, CA, USA), and intubated with a 16-gauge blunt polycarbonate catheter. Then 500 μL of NP suspension or vehicle control (PBS with 3 % rat serum) was instilled once using a sterile syringe.

### OVA-induced allergic airway inflammation model

As a positive control for IgE-mediated allergic inflammation, the OVA-induced allergic airway inflammation model was established according to the previously described method, with slight modification [35]. Briefly, 6-week old female Wistar rats were sensitized on days 0, 7, and 14 with an intraperitoneal injection of 1 mg OVA (Grade V, Sigma-Aldrich, St Louis, MO, USA) and 100 mg aluminum hydroxide (Sigma-Aldrich) in 1 mL sterile saline. On day 21, rats were challenged with 1 mg OVA by intratracheal instillation and then sacrificed on day 22 to collect the BALF and serum.

### Intratracheal instillation of nickel ion (NiCl_2_)

Because we hypothesized that NiO NPs can produce eosinophilia via the accumulation of nickel ions in the phagolysosomes of phagocytes, NiCl_2_ were instilled into lungs of rats to demonstrate the direct effect of nickel ions. Our previous studies also showed that instillation CoCl_2_ and ZnCl_2_ produced similar magnitude of eosinophilia as observed in CoO and ZnO at an equal metal concentrations [8, 11]. Although NiO NPs in the lung might not completely dissolved in the lung within 3 days after instillation, we selected the dose of NiCl_2_ at 378.0 μg/rat (171.1 μg Ni/rat), which is equivalent nickel concentration of 200 cm^2^/rat of NiO NPs to evaluate whether the dissolved nickel from NiO NPs directly induce pulmonary eosinophilia or not. NiCl_2_ (Sigma-Aldrich) dissolved in sterile Ca^2+^- and Mg^2+^-free PBS (Life Technologies) was intratracheally instilled into the lungs of rats and then sacrificed at 24 h post-instillation to collect the BALF and serum.

### Preparation of BALF

At each time point, rats were anesthetized by isoflurane inhalation (Piramal Critical Care) and euthanized by removing blood from the inferior vena cava. The trachea was then cannulated by 14-gauge blunt stainless-steel needle and lavaged in-situ with 8 mL sterile cold Ca^2+^- and Mg^2+^-free PBS (Life Technologies) 4 times. The recovery volumes of the first lavage were about 7 mL and the subsequent lavages were about 8 mL. The first lavage was collected separately, and the cell-free supernatant was kept at −70 °C for further assays. Cell pellets from 4 lavages were pooled for total cell counts and cytological evaluation. The total number of cells was counted using a NucleoCounter (Chemometec, Allerod, Denmark); 4 × 10^4^ cells were attached to glass slides by spinning at 27 *g* for 5 min using a cytospin (Hanil, Seoul, Korea). Slides were then dried, fixed with 100 % methanol, and stained with Diff-Quik (Thermo Fisher Scientific, Waltham, MA, USA). Differential cell counting was performed under a light microscope based on the morphology of cells. A minimum of 300 cells per slide was counted.

### Measurement of nickel levels in BALF

The levels of nickel in BALF were measured to understand the uptake and dissolution of NiO NPs in the lung. The cell-free first lavage was centrifuged at 15000 *g* for 30 min to collect NP-free supernatant. Then, the concentration of dissolved nickel ions in the supernatant was measured using inductively coupled mass spectrometry (ICP-MS; Agilent Technologies).

### Measurement of LDH, total protein, and pro-inflammatory cytokines in BALF

The levels of LDH, a maker for cytotoxicity, were measured in BALF using an LDH assay kit (Roche Diagnostics, Mannheim, Germany). Total protein, a maker for vascular permeability, was measured in BALF using a bicinchoninic acid (BCA) assay kit (Thermo Fisher Scientific). To evaluate the underlying mechanism of inflammation by NiO NPs, pro-inflammatory cytokines related to acute neutrophilic inflammation (IL-1β and CINC-3), eosinophilic inflammation (eotaxin and IL-4), and Th1 response (IFN-γ) were measured in BALF using a Duoset enzyme-linked immunosorbent assay (ELISA) kit (all from R&D systems, Minneapolis, MN, USA).

### Measurement of total IgE, C3a, and C5a in BALF and serum

To evaluate the mechanism of allergic lung inflammation, the level of total IgE was measured in BALF and serum using an ELISA kit (Komabiotech, Seoul, Korea) according to the manufacturer’s instruction. The levels of C3a and C5a, marker for complement activation, were measured in BALF and serum using a C3a ELISA kit (MBS2503333, MyBioSource, San Diego, CA, USA) and C5a ELISA kit (MBS2506864, MyBioSource).

### Measurement of intracellular eotaxin in alveolar macrophages and normal lung tissue

To evaluate whether the direct lysis of cells can release intracellular eotaxin, the levels of eotaxin were measured using alveolar macrophages and normal lung tissue before and after addition of cell lysis buffer. To collect the alveolar macrophages, 6-week old female Wistar rats (Samtako) were sacrificed and lungs were lavaged 4 times with 8 mL of PBS and cell pellets were collected by simple centrifugation and suspended with 1 mL of PBS. The number of total cells was counted using a NucleoCounter (Chemometec) and cytospin slides were prepared to confirm that more than 99 % of cells in BALF are alveolar macrophages. Then, cell lysis buffer (Atto, Tokyo, Japan) was added at a volume of 1 mL for 1 × 10^7^ cells. To evaluate the dose–response relationship, the cells were lysed at different concentrations of cell lysis buffer by serial dilution with PBS. On the other hand, normal lung tissue was collected from 6-week old female Wistar rats (Samtako) and homogenized using tissue homogenizer. Then, cell lysis buffer (Atto) was added at a volume of 1 mL for 50 mg of lung tissue. Cell lysis buffer was serially diluted with PBS to evaluate the dose–response relationship. Alveolar macrophages and tissue homogenates were incubated with cell lysis buffer for 30 min and cell-free supernatants were collected by centrifugation at 500 *g* for 5 min followed by centrifugation at 15000 *g* for 5 min. The levels of LDH and eotaxin levels were then measured using a LDH assay kit (Roche Diagnostics) and a Duoset ELISA kit (R&D systems), respectively.

### Statistical analysis

Number of rats for NiO NPs was 10 per group, while the number of rats for NiCl_2_ and OVA-induced airway inflammation group was 4 per group. One-way analysis of variance (ANOVA) was applied to the data having more than 3 groups, while the unpaired student’s *t*-test was applied to the OVA-induced airway inflammation group and NiCl_2_ treated group. When ANOVA test showed statistical significance (*p* < 0.05), each group was compared using post-hoc Tukey’s pairwise comparisons. Pearson correlation test was applied to evaluate the correlation between the levels of LDH and eotaxin in cell lysis experiment. All statistical analysis and graphs were prepared using GraphPad Prism (version 6.0 for Windows; GraphPad Software, San Diego, CA, USA). A value of *p* < 0.05 was considered statistically significant.

## Abbreviations

ANOVA, analysis of variance; BALF, bronchoalveolar lavage fluid; BCA, bicinchoninic acid; BET, Brunauer, Emmett, and Teller; CINC-3, cytokine-induced neutrophil chemoattractant-3; CoCl_2_, cobalt chloride; CoO, cobalt monoxide; CuO, copper oxide; DW, distilled water; ELISA, enzyme-linked immunosorbent assay; Fe_2_O_3_, iron oxide; FE-SEM, field emission-type scanning electron microscopy; ICP-MS, inductively coupled mass spectrometry; IFN-γ, interferon-γ; IgE, immunoglobulin E; IL, interleukin; LAL, *Limulus* Amoebocyte Lysate; LDH, lactate dehydrogenase; Ni, nickel; NiCl_2_, nickel chloride; NiO, nickel oxide; NP, nanoparticle; OVA, ovalbumin; PBS, phosphate buffered saline; PM, particulate matter; ROS, reactive oxygen species; TEM, transmission electron microscopy; Th1-type, T helper type 1; TiO_2_, titanium dioxide; ZnCl_2_, zinc chloride; ZnO, zinc oxide

## Additional file

Additional file 1:
**Figure S1.** Transmission electron microscopy (TEM) and field emission-type scanning electron microscopy (FE-SEM) image of NiO NPs. (A) TEM. (B) FE-SEM. **Figure S2.** Cytological analysis of BALF after intratracheal instillation of NiO NPs. NiO NPs were instilled at 50 and 100 cm^2^/rat and cytological evaluation was performed at 1, 2, 3, and 4 days after instillation. (A), Number of total cells. (B) Number of macrophages. (C) Number of neutrophils. (D) Number of eosinophils. Mean ± SEM (*n* = 4). One-way ANOVA test was applied for comparison between NiO NPs and vehicle control (VEH) with statistical significance indicated by ^*^ *p* < 0.05, ^**^ *p* < 0.01, and ^***^ *p* < 0.001. **Figure S3.** The levels of nickel (Ni) in BALF at 1, 2, 3, and 4 days after intratracheal instillation of NiO NPs at 200 cm^2^/rat. Mean ± SEM (*n* = 4). One-way ANOVA test was applied for comparison between NiO NPs and vehicle control (VEH) with statistical significance indicated by ^*^ *p* < 0.05 and ^**^ *p* < 0.01. **Figure S4.** Levels of lactate dehydrogenase (LDH) and total protein in BALF treated with NiO NPs. NiO NPs were instilled at 50 and 100 cm^2^/rat and the levels of LDH and total protein were measured at 1, 2, 3, and 4 days after instillation. (A), Levels of LDH. (B), Levels of total protein. Mean ± SEM (*n* = 4). One-way ANOVA test was applied for comparison between NiO NPs and vehicle control (VEH) with statistical significance indicated by ^***^ *p* < 0.001. (DOCX 725 kb)
