# Supplementary material for: Nickel oxide nanoparticles can recruit eosinophils in the lungs of rats by the direct release of intracellular eotaxin
Source: Part Fibre Toxicol. 2016 Jun 9;13:30. doi: 10.1186/s12989-016-0142-8 (PMC4899890; doi:10.1186/s12989-016-0142-8)
Supplement: Additional file 1: — Figure S1. Transmission electron microscopy (TEM) and field emission-type scanning electron microscopy (FE-SEM) image of NiO NPs. (A) TEM. (B) FE-SEM. Figure S2. Cytological analysis of BALF after intratracheal instillation of NiO NPs. NiO NPs were instilled at 50 and 100 cm2/rat and cytological evaluation was performed at 1, 2, 3, and 4 days after instillation. (A), Number of total cells. (B) Number of macrophages. (C) Number of neutrophils. (D) Number of eosinophils. Mean ± SEM (n = 4). One-way ANOVA test was applied for comparison between NiO NPs and vehicle control (VEH) with statistical significance indicated by * p < 0.05, ** p < 0.01, and *** p < 0.001. Figure S3. The levels of nickel (Ni) in BALF at 1, 2, 3, and 4 days after intratracheal instillation of NiO NPs at 200 cm2/rat. Mean ± SEM (n = 4). One-way ANOVA test was applied for comparison between NiO NPs and vehicle control (VEH) with statistical significance indicated by * p < 0.05 and ** p < 0.01. Figure S4. Levels of lactate dehydrogenase (LDH) and total protein in BALF treated with NiO NPs. NiO NPs were instilled at 50 and 100 cm2/rat and the levels of LDH and total protein were measured at 1, 2, 3, and 4 days after instillation. (A), Levels of LDH. (B), Levels of total protein. Mean ± SEM (n = 4). One-way ANOVA test was applied for comparison between NiO NPs and vehicle control (VEH) with statistical significance indicated by *** p < 0.001. (DOCX 725 kb) [file 12989_2016_142_MOESM1_ESM.docx]

Supporting Information





Figure S1. Transmission electron microscopy (TEM) and field emission-type scanning electron microscopy (FE-SEM) image of NiO NPs. (A) TEM. (B) FE-SEM.


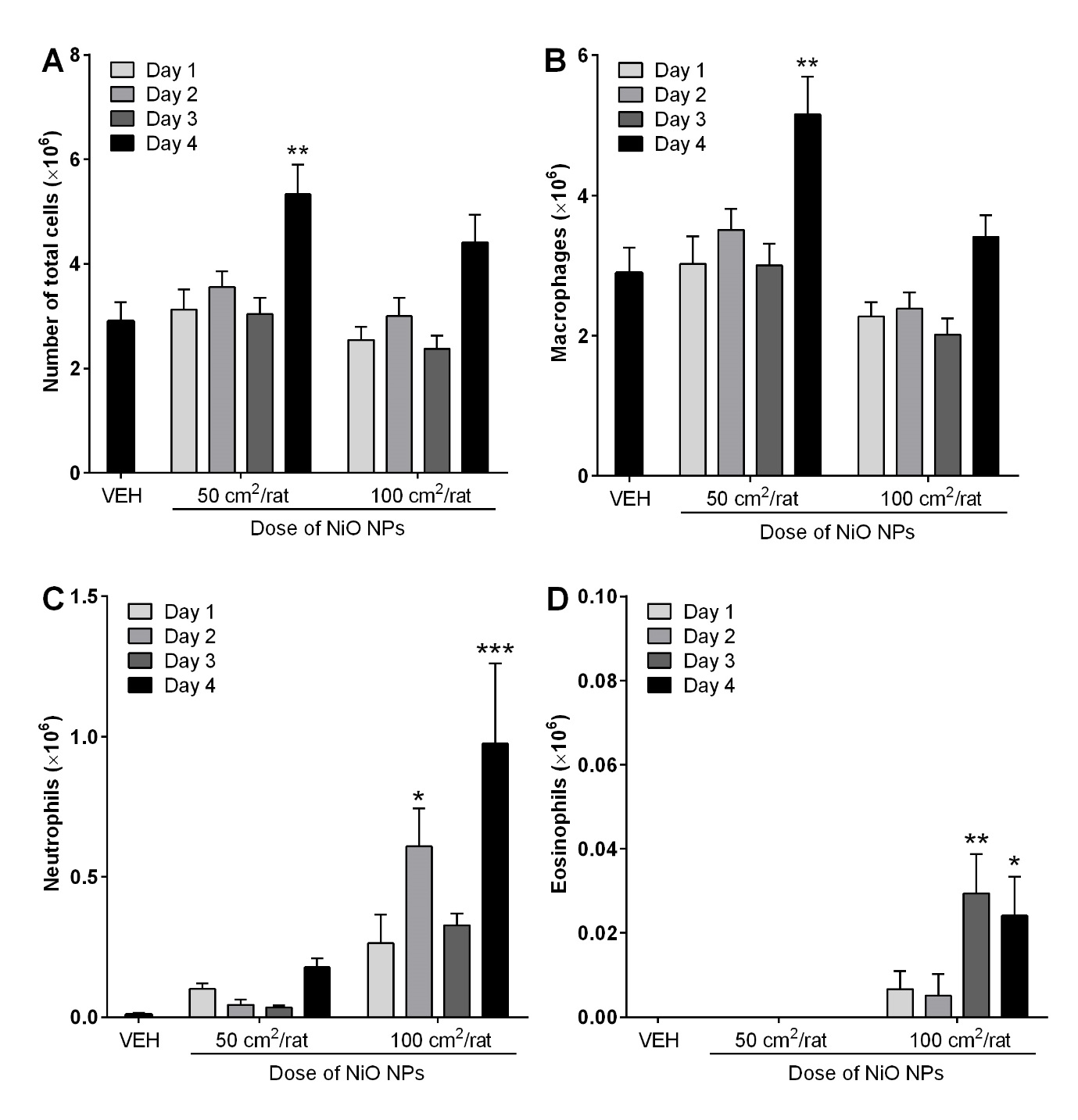


Figure S2. Cytological analysis of BALF after intratracheal instillation of NiO NPs. NiO NPs were instilled at 50 and 100 cm^2^/rat and cytological evaluation was performed at 1, 2, 3, and 4 days after instillation. (A), Number of total cells. (B) Number of macrophages. (C) Number of neutrophils. (D) Number of eosinophils. Mean ± SEM (*n*=4). One-way ANOVA test was applied for comparison between NiO NPs and vehicle control (VEH) with statistical significance indicated by ^*^*p*<0.05, ^**^*p*<0.01, and ^***^*p*<0.001.


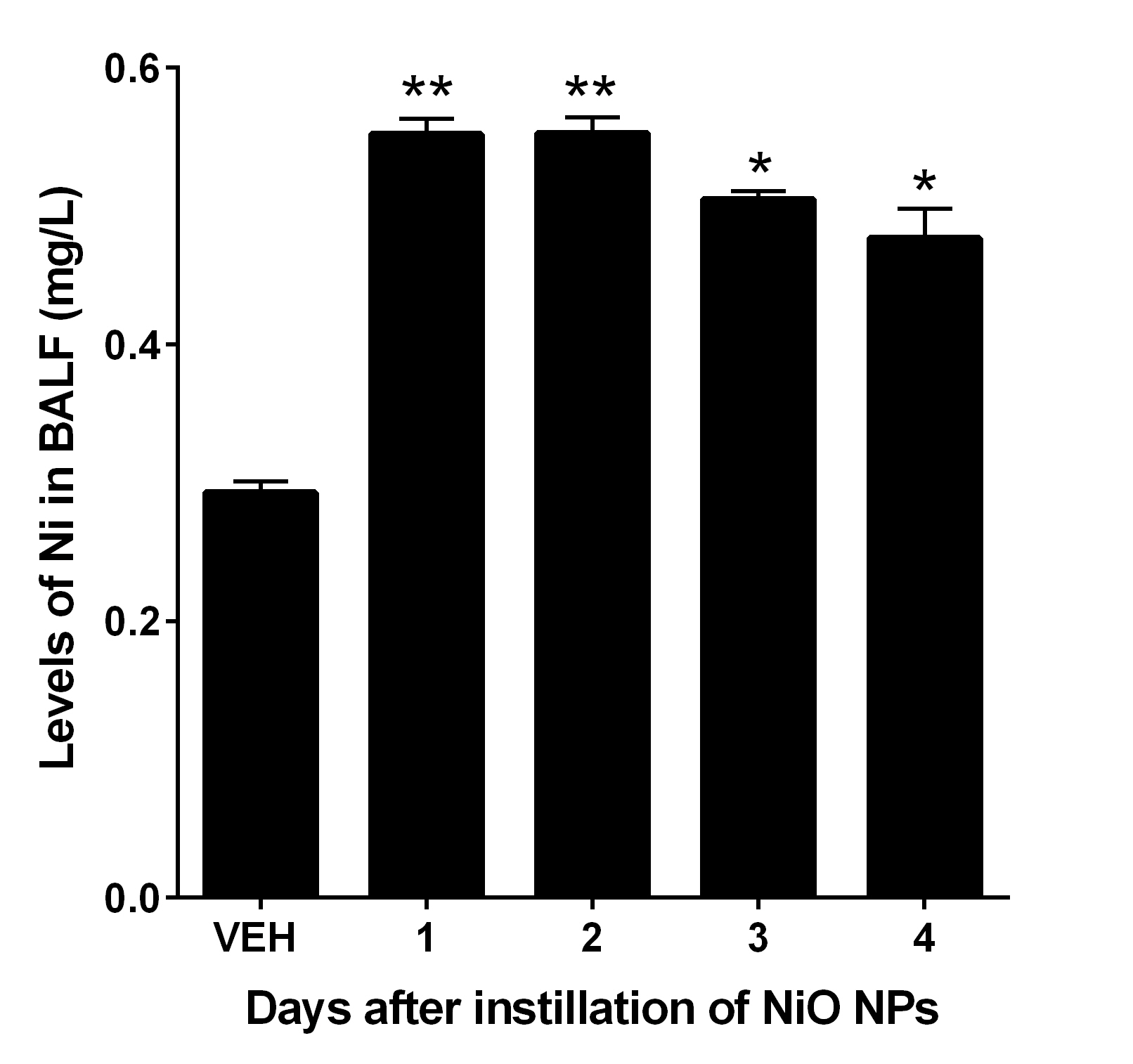


Figure S3. The levels of nickel (Ni) in BALF at 1, 2, 3, and 4 days after intratracheal instillation of NiO NPs at 200 cm^2^/rat. Mean ± SEM (*n*=4). One-way ANOVA test was applied for comparison between NiO NPs and vehicle control (VEH) with statistical significance indicated by ^*^*p*<0.05 and ^**^*p*<0.01.


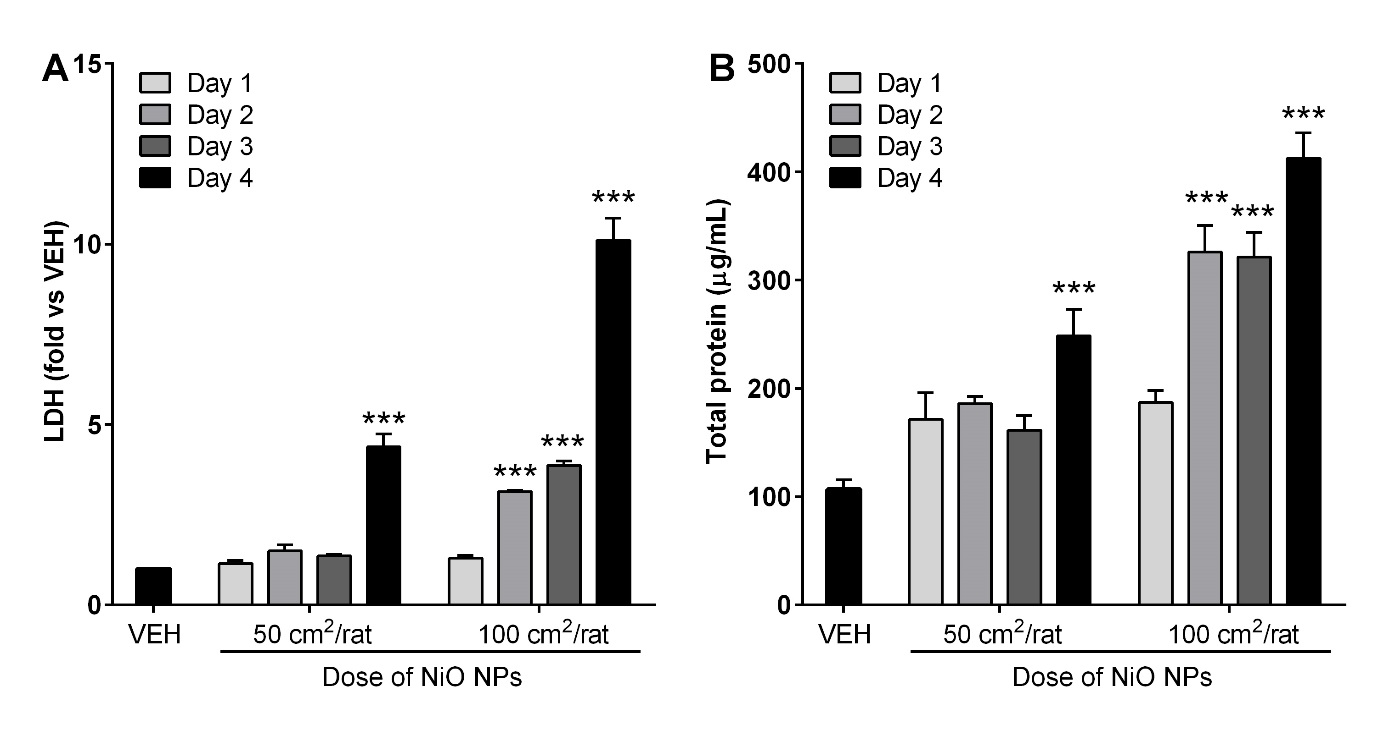


Figure S4. Levels of lactate dehydrogenase (LDH) and total protein in BALF treated with NiO NPs. NiO NPs were instilled at 50 and 100 cm^2^/rat and the levels of LDH and total protein were measured at 1, 2, 3, and 4 days after instillation. (A), Levels of LDH. (B), Levels of total protein. Mean ± SEM (*n*=4). One-way ANOVA test was applied for comparison between NiO NPs and vehicle control (VEH) with statistical significance indicated by ^***^*p*<0.001.
